# Guillain-Barre syndrome following COVID-19 vaccines: A review of literature

**DOI:** 10.3389/fimmu.2023.1078197

**Published:** 2023-02-15

**Authors:** Miao Yu, Shuang Nie, Yue Qiao, Ying Ma

**Affiliations:** ^1^ Department of Neurology, Shengjing Hospital of China Medical University, Shenyang, China; ^2^ Department of Neurology, Shenyang First People’s Hospital, Shenyang, China

**Keywords:** GBS, Guillain-Barre syndrome, Miller Fisher syndrome, COVID-19 vaccination, SARS-CoV-2

## Abstract

**Objective:**

This study aimed to retrospectively analyze reported Guillain–Barré syndrome (GBS) cases that occurred after COVID-19 vaccination.

**Methods:**

Case reports of GBS following COVID-19 vaccination that were published before May 14, 2022, were retrieved from PubMed. The cases were retrospectively analyzed for their basic characteristics, vaccine types, the number of vaccination doses before onset, clinical manifestations, laboratory test results, neurophysiological examination results, treatment, and prognosis.

**Results:**

Retrospective analysis of 60 case reports revealed that post-COVID-19 vaccination GBS occurred mostly after the first dose of the vaccination (54 cases, 90%) and was common for DNA vaccination (38 cases, 63%), common in middle-aged and elderly people (mean age: 54.5 years), and also common in men (36 cases, 60%). The mean time from vaccination to onset was 12.3 days. The classical GBS (31 cases, 52%) was the major clinical classification and the AIDP subtype (37 cases, 71%) was the major neurophysiological subtype, but the positive rate of anti-ganglioside antibodies was low (7 cases, 20%). Bilateral facial nerve palsy (76% vs 18%) and facial palsy with distal paresthesia (38% vs 5%) were more common for DNA vaccination than for RNA vaccination.

**Conclusion:**

After reviewing the literature, we proposed a possible association between the risk of GBS and the first dose of the COVID-19 vaccines, especially DNA vaccines. The higher rate of facial involvement and a lower positive rate of anti-ganglioside antibodies may be a characteristic feature of GBS following COVID-19 vaccination. The causal relationship between GBS and COVID-19 vaccination remains speculative, more research is needed to establish an association between GBS and COVID-19 vaccination. We recommend surveillance for GBS following vaccination, because it is important in determining the true incidence of GBS following COVID-19 vaccination, as well as in the development of a more safer vaccine.

## Introduction

With large-scale COVID-19 vaccination happening worldwide, there are increasing reports of neurological complications after COVID-19 vaccination, such as transverse myelitis, multiple sclerosis, and facial palsy (1). Guillain–Barré syndrome (GBS) is also one of the neurological complications, with Waheed et al. reporting the first case of GBS after the first dose of Pfizer COVID-19 vaccination in February 2021 (2). Clinical trials in the Americas have also demonstrated that COVID-19 vaccines involving viral genetic material may trigger GBS (3–5).

Since there are no specific medicines for COVID-19, vaccination remain the only weapons to fight this deadly disease. But the vaccination proved to be a double-edged, identifying the side effects of COVID-19 vaccination is equally important. Severe neurological adverse events is defined as a post-vaccination event that is either life-threatening, requires hospitalization, or results in severe disability. GBS is one of the serious neurologic adverse events, which may be the major source of vaccine hesitancy. However, the relationship between COVID-19 vaccination and GBS, actually described only in single case reports. It is important to review these cases to better understand whether a possible association between COVID-19 vaccination and GBS actually exists, and how likely is it that the COVID-19 vaccination will cause GBS? In this regard, we reviewed the GBS cases following COVID-19 vaccination, aimed at revealing whether this syndrome is associated with COVID-19 vaccination and exploring possible pathogenic mechanisms that lead to GBS by assessing different demographic, clinical, and neurophysiological aspects of cases with GBS following COVID-19 vaccination.

## Methods

**Figure 1:**
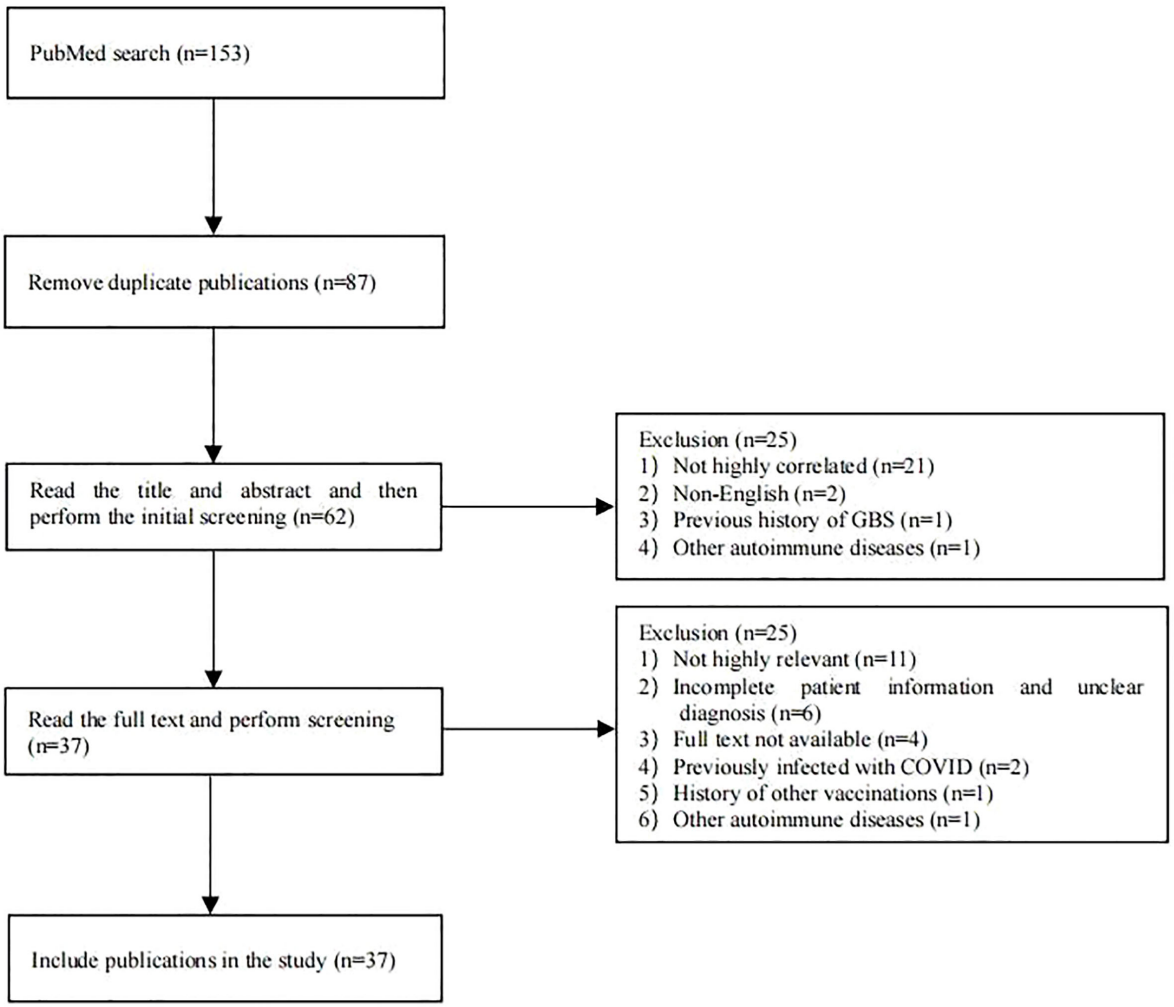
Flow chart of literature search and screening.

PubMed was searched for case reports published before May 14, 2022, using the search terms “COVID-19,” “SARS-CoV-2,” “vaccination” or “vaccine,” “Guillain–Barré syndrome,” “GBS,” “Miller Fisher syndrome,” “MFS,” “AIDP,” “AMAN,” “AMSAN,” “acute ataxic neuropathy,” “acute ophthalmoparesis,” “pharyngeal cervical brachial,” “polyneuritis cranialis,” or “bilateral facial weakness with paresthesia.” Exclusion criteria were: 1) case reports with a history of COVID-19 infection or other infections prior to onset, 2) case reports with a history of other vaccinations prior to onset, or 3) case reports with a history of GBS or other autoimmune abnormalities. The flow chart for the literature search and screening is shown in **Figure 1**. Detailed information was collected for each case using a pre-designed form, including general demographic data, type of vaccination, the number of vaccination doses before onset, clinical manifestations, laboratory test results of cerebrospinal fluid (CSF), electrophysiological examination results, treatment options, and prognosis. The accuracy of GBS and MFS diagnosis in the case reports was assessed according to the Brighton Collaboration GBS Case Classification (6).

All statistical analyses were performed using SPSS 26.0. Continuous variables are expressed as mean ± standard deviation (SD). All categorical variables were expressed as frequencies (percentages). Differences between measurement data were assessed using independent t-test, and differences between count data were assessed using the chi-squared test, with p 0.05 indicating significant differences.

## Results

### Literature review

A total of 87 publications were retrieved from the PubMed database. After screening, 37 publications were included, consisting of 60 cases of post-COVID-19 vaccination GBS (**Figure 1**), with their general information, type of vaccination, the number of vaccination doses before onset, clinical manifestations, laboratory test results, electrophysiological examination results, treatment options, and prognosis; these details are summarized in **Table 1** (2, 5, 7–41).

**Table 1:** Case reports of patients with GBS who associated with COVID-19 vaccination.

Reference	Country	Sex/Age,y	Type of vaccine(Dose1st/2nd;DNA/RNA)	Vaccination to symptom onset(days)	Clinical presentation	CSF WBC (/μl)	CSF protein (mg/dl)	Antiganglioside antibody	EMG	Clinical classification	Treatment	Outcome
McKean et al. (2021)	Malta	M/48	ChAdOx1 (1st;DNA)	10	Distal paraesthesia, quadriparesis,facial diplegia,back pain	8	127	Negative	AIDP	Classic GBS	IVIG+Seroids	PR
Trimboli et al. (7)	Italy	F/25	BNT162b2 (2nd;RNA)	5	Distal paraesthesia, paraparesis	NA(albuminocytological dissociation)		NA	AIDP	Paraparetic	IVIG	PR
Scendoni et al. (8)	Italy	F/82	BNT162b2 (2nd;RNA)	14	Paraesthesia, ascending paralysis, decreased deep tendon reflexes	2	570	Anti-sulfatide IgG and IgM , anti-GM2 IgM , anti-GM4 IgM	AMSAN	Classic GBS	IVIG	PR
Introna et al. (9)	Italy	M/62	ChAdOx1 (1st;DNA)	10	Distal paresthesia, facial diplegia, quadriparesis, decreased deep tendon reflexes, bulbar palsy	5	101	Anti-GM1 IgG	AIDP	Classic GBS	IVIG	PR
Nasuelli et al. (11)	Italy	M/59	ChAdOx1 (1st;DNA)	10	Distal paresthesia, facial diplegia	Normal	140	Negative	AIDP	Bilateral facial palsy with paresthesia	IVIG	PR
Čenščák et al. (12)	Czech Republic	M/42	BNT162b2 (1st;RNA)	14	Distal paraesthesia, quadriparesis	6	244	Negative	AIDP	Classic GBS	IVIG	PR
Hasan et al. (41)	UK	F/62	ChAdOx1 (1st;DNA)	11	Distal paraesthesia, quadriparesis, decreased deep tendon reflexes	1	90	NA	AIDP	Classic GBS	IVIG+MV	PR
Kanabar et al. (13)	UK	M/56	ChAdOx1 (1st;DNA)	7	Distal paresthesia, facial diplegia,back pain	2	160	NA	AIDP	Bilateral facial palsy with paresthesia	IVIG	CR
Patel et al. (14)	UK	M/37	ChAdOx1 (1st;DNA)	14	Distal paresthesia,quadriparesis,back pain	0	177	Negative	NA	Classic GBS	IVIG	PR
Allen et al. (40)	UK	M/54	ChAdOx1 (1st;DNA)	12	Distal paresthesia, facial diplegia	19	163	Negative	Normal(limb)	Bilateral facial palsy with paresthesia	Seroids	NA
		M/20	ChAdOx1 (1st;DNA)	21	Distal paresthesia, facial diplegia	14	123	Negative	Normal(limb)	Bilateral facial palsy with paresthesia	Seroids	NA
		M/57	ChAdOx1 (1st;DNA)	11	Distal paresthesia, facial diplegia,back pain,bulbar palsy	8	247	Negative	Normal(limb)	Bilateral facial palsy with paresthesia	IVIG	NA
		M/55	ChAdOx1 (1st;DNA)	22	Paresthesia,facial diplegia	4	89	Negative	NA	Bilateral facial palsy with paresthesia	None	NA
Bonifacio et al. (39)	UK	M/66	ChAdOx1 (1st;DNA)	7	Paresthesia,facial diplegia,back pain	2	199	Negative	AIDP	Bilateral facial palsy with paresthesia	IVIG	PR
		M/43	ChAdOx1 (1st;DNA)	11	Paresthesia,facial diplegia	23	281	Negative	AIDP	Bilateral facial palsy with paresthesia	IVIG	PR
		M/51	ChAdOx1 (1st;DNA)	7	Paresthesia,facial diplegia	1	514	Anti-GM3;Anti-GM4	AIDP	Bilateral facial palsy with paresthesia	None	PR
		F/71	ChAdOx1 (1st;DNA)	12	Paresthesia,facial diplegia,back pain	1	96	Negative	AIDP	Bilateral facial palsy with paresthesia	None	PR
		M/53	ChAdOx1 (1st;DNA)	8	Paresthesia,facial diplegia	0	122	Negative	NA	Bilateral facial palsy with paresthesia	None	PR
Azam et al. (15)	UK	M/67	ChAdOx1 (1st;DNA)	15	Quadriparesi, facial diplegia	0	390	Negative	AIDP	Classic GBS	IVIG	PR
Rossetti et al. (38)	USA	M/38	Ad26.COV2.S (1st;DNA)	32	Distal paresthesia, facial diplegia, bulbar palsy, ophthalmoplegia	7	181	NA	AIDP	Bilateral facial palsy with paresthesia	IVIG	PR
Waheed et al. (2)	USA	F/82	BNT162b2 (1st;RNA)	14	Paresthesia, paraparesis,body pain	4	88	NA	NA	Paraparetic	IVIG	NA
Loza et al. (2021)	USA	F/60	Ad26.COV2.S (1st;DNA)	17	Paraparesis, facial diplegia, paresthesia, ophthalmoplegia, decreased deep tendon reflexes	9	140	Negative	AIDP	GBS / MFS overlap variants	IVIG	PR
Ogbebor et al. (17)	USA	F/86	BNT162b2 (1st;RNA)	1	Paraparesis	2	162	NA	NA	Paraparetic	IVIG	PR
Prasad et al. (18)	USA	M/41	Ad26.COV2.S (1st;DNA)	15	Distal paresthesia, facial diplegia, ataxia	50	562	NA	AIDP	Bilateral facial palsy with paresthesia	IVIG	PR
Malamud et al. (37)	USA	M/14	BNT162b2 (2nd;RNA)	30	Quadriparesis,facial diplegia,back pain, decreased deep tendon reflexes	4	165	Negative	AIDP	Classic GBS	IVIG	PR
Thant et al. (19)	USA	F/66	Ad26.COV2.S (1st;DNA)	14	Paresthesia, quadriparesis	Normal	75	NA	NA	Classic GBS	IVIG+PE	PR
Min et al. (20)	Korea	M/58	ChAdOx1 (1st;DNA)	3	Distal paresthesia (clinically pure sensory)	2	70	Negative	AIDP	Sensory GBS	Gabapentin	PR
		F/37	ChAdOx1 (1st;DNA)	4	Distal paresthesia (clinically pure sensory)	NA	NA	Negative	Normal	Sensory GBS	Gabapentin	PR
Kim N et al. (21)	Korea	M/21	BNT162b2 (1st;RNA)	21	Quadriparesis, paresthesia, facial diplegia, ataxia	Normal	73	Negative	AIDP	Classic GBS	IVIG	PR
Kim JW et al. (22)	Korea	M/42	ChAdOx1 (1st;DNA)	14	Facial diplegia , quadriparesis	3	62	Negative	AIDP	Classic GBS	IVIG+PE+MV	PR
		F/48	BNT162b2 (1st;RNA)	14	Quadriparesis, facial diplegia, paresthesia	1	150	Negative	AIDP	Classic GBS	IVIG	PR
Biswas et al. (23)	India	F/49	ChAdOx1 (1st;DNA)	7	Quadriparesis, facial diplegia	NA	NA	NA	AIDP	Classic GBS	IVIG+Seroids	PR
James J et al. (24)	India	M/60	ChAdOx1 (1st;DNA)	11	Quadriparesis,facial diplegia,paresthesia	0	149	NA	AMSAN	Classic GBS	IVIG	PR
		M/66	ChAdOx1 (1st;DNA)	12	Quadriparesis, paresthesia	0	84	NA	AIDP	Classic GBS	IVIG+Seroids	PR
		F/54	ChAdOx1 (1st;DNA)	13	Quadriparesis,paresthesia,bulbar palsy	NA	NA	Negative	AIDP	Classic GBS	IVIG+Seroids	PR
Marammatom et al. (25)	India	F/43	ChAdOx1 (1st;DNA)	10	Quadriparesis,facial diplegia, back pain	2	85	NA	AIDP	Classic GBS	IVIG+MV	CR
		F/67	ChAdOx1 (1st;DNA)	14	Quadriparesis, distal paresthesia, facial diplegia,	3	346	Negative	AMSAN	Classic GBS	IVIG+PE+MV	Bed- bound
		F/53	ChAdOx1 (1st;DNA)	12	Quadriparesis, facial diplegia, back pain	3	120	Negative	AIDP	Classic GBS	IVIG+MV	PR
		F/68	ChAdOx1 (1st;DNA)	14	Quadriparesis, paresthesia, facial diplegia,back pain, bulbar palsy	4	75	Negative	AIDP	Classic GBS	IVIG+MV	PR
		M/70	ChAdOx1 (1st;DNA)	11	Quadriparesis, facial diplegia, paresthesia,bulbar palsy	NA	NA	NA	AIDP	Classic GBS	IVIG+MV	PR
		F/69	ChAdOx1 (1st;DNA)	12	Quadriparesis,facial diplegia,paresthesia	NA	NA	NA	AIDP	Classic GBS	IVIG+PE	PR
		F/69	ChAdOx1 (1st;DNA)	13	Quadriparesis,facial diplegia,paresthesia	2	83	NA	AIDP	Classic GBS	IVIG+MV	PR
Tutar NK et al. (36)	Turkey	M/76	CoronaVac (2nd;DNA)	5	Myalgia, paralysis	NA(albuminocytological dissociation)		Negative	AMSAN	Paraparetic	IVIG	PR
Theuriet J et al. (26)	France	M/72	ChAdOx1 (1st;DNA)	16	Distal dysesthesia , ascending paralysis, facial diplegia	Normal	62	Anti-GM3 IgM	AIDP	Classic GBS	IVIG	NA
Bouattour et al. (27)	Tunisia	M/67	BNT162b2 (1st;RNA)	3	Quadriparesis	4	80	Negative	AIDP	Paraparetic	IVIG	CR
Razok et al. (28)	Qatar	M/73	BNT162b2 (2nd;RNA)	15	Paraparesis	NA	80	NA	AIDP	Paraparetic	IVIG	PR
Garcia-Grimshaw M et al. (29)	Mexico	M/33	BNT162b2 (1st;RNA)	28	Facial diplegia	0	67	NA	AIDP	Bilateral facial palsy with paresthesia	IVIG	PR
		M/25	BNT162b2 (1st;RNA)	12	Distal paresthesia, quadriparesis	20	64	NA	AIDP	Classic GBS	IVIG	PR
		F/53	BNT162b2 (1st;RNA)	6	Quadriparesis	0	15	NA	AMAN	Classic GBS	IVIG+MV	PR
		M/72	BNT162b2 (1st;RNA)	4	Quadriparesis	NA	NA	NA	AMAN	Classic GBS	IVIG	PR
		M/31	BNT162b2 (1st;RNA)	11	Quadriparesis	NA	NA	NA	AIDP	Classic GBS	IVIG	PR
		F/67	BNT162b2 (1st;RNA)	4	Quadriparesis	22	30	NA	AMAN	Classic GBS	IVIG+MV	Dead
		F/81	BNT162b2 (1st;RNA)	3	Quadriparesis	0	414	NA	AIDP	Classic GBS	IVIG	PR
Abičić et al. (5)	Croatia	F/24	BNT162b2 (1st;RNA)	18	Ophthalmoplegia	2	30	Anti-GQ1b	Normal	MFS	IVIG	PR
Dang et al. (30)	Australia	M/63	ChAdOx1 (1st;DNA)	14	Facial diplegia, quadriparesis,ataxia, ophthalmoplegia	5	299	Negative	AMSAN	GBS / MFS overlap variants	IVIG	PR
Shao et al. (31)	China	M/41	ChAdOx1 (1st;DNA)	7	Paresthesia,facial diplegia	Normal	160	NA	NA	Bilateral facial palsy with paresthesia	IVIG	PR
Fukushima et al. (32)	Japan	F/55	BNT162b2 (1st;RNA)	13	Paresthesia	1	44.5	Anti-GM1	NA	Sensory GBS	IVIG	PR
Yamakawa et al. (33)	Japan	M/30	BNT162b2 (2nd;RNA)	7	Ophthalmoplegia,ataxic, absent tendon reflexes	Normal	Normal	Anti-GQ1b,Anti-GT1a	Normal	MFS	IVIG	PR
Nishiguchi et al. (34)	Japan	M/71	BNT162b2 (1st;RNA)	18	Ataxia,ophthalmoplegia	1	67	Negative	Normal	MFS	IVIG	CR
Takahashi et al. (35)	Japan	F/65	BNT162b2 (1st;RNA)	24	Quadriparesis	1	38	Negative	AIDP	Classic GBS	IVIG+MV	PR

**Figure 2:**
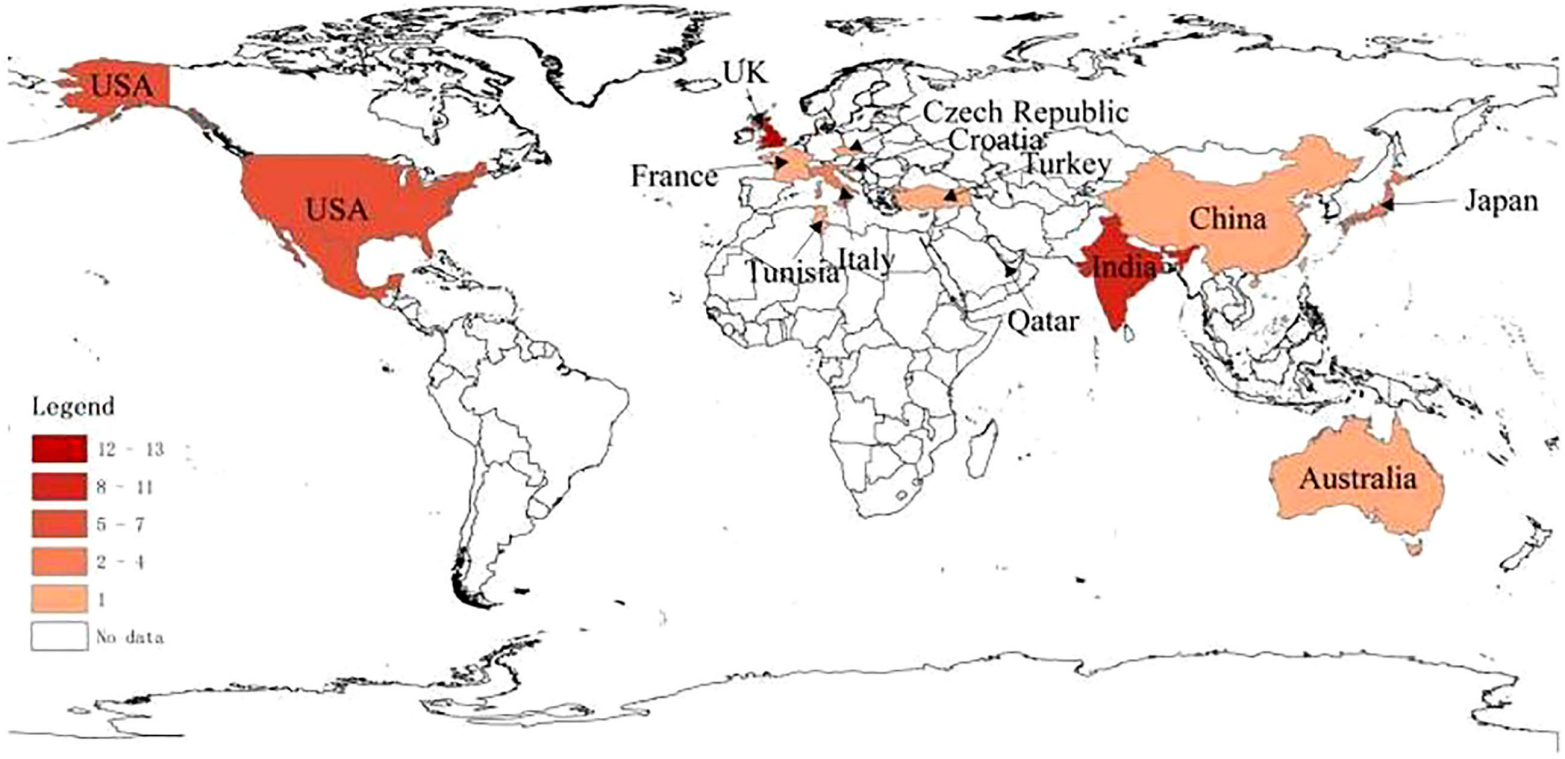
Geographic distribution of cases of Guillain–Barre syndrome after COVID-19 vaccination.

F, female; M, male;CSF, cerebral spinal fluid; WBC, white blood cell; EMG, electromyography; IVIG, Intravenous immunoglobulin; MV, mechanical ventilation; PE, plasma exchange; PR, partial recovery; CR, complete recovery; NA, not available; ; GBS, Guillain-Barré Syndrome; MFS, Miller-Fisher Syndrome; AIDP, acute inflammatory demyelinating polyneuropathy; AMAN, acute motor axonal neuropathy; AMSAN, acute motor and sensory axonal neuropathy;

Geographical distribution of all cases (**Figure 2**) was as follows: 13 cases in the UK, 11 cases in India, 7 cases in Mexico, 7 cases in the US, 5 cases in Korea, 4 cases in Italy, 4 cases in Japan, 1 case each in Croatia, Australia, China, Malta, Turkey, France, Tunisia, Qatar, and the Czech Republic.

Patient age in the cases ranged 14–86 years with a mean age of 54.5 years; there were 36 male cases (60%) and 24 female cases (40%), with a male-to-female ratio of 1.5:1. DNA vaccines were the major vaccine type, provided in 37 (62%) cases, consisting of 33 (55%) vaccinated with ChAdOx1-S, 4 (7%) vaccinated with Ad26.COV2S, followed by RNA vaccine BNT162b2 in 22 (37%) cases, and CoronaVac in 1 case (2%). Symptoms occurred in 54 (90%) cases after the first dose of vaccine and in only 6 (10%) cases after the second dose of vaccine. The mean time from vaccination to symptom onset was 12.3 days (1–32 days). The initial symptoms of GBS, sorted in order of decreasing frequency, were limb weakness in 39 (65%) cases, paresthesia in 38 (63%) cases, and bilateral facial nerve palsy in 34 (57%) cases. Fifty-three cases underwent lumbar puncture, of whom 47 (89%) had CSF albuminocytologic dissociation. Thirty-five cases underwent tests for anti-ganglioside antibodies, but only 7 (20%) tested positive as follows: (1) n=1 for all of anti-sulfatide antibodies, anti-GM2 antibodies, and anti-GM4 antibodies; (2) n=1 for anti-GM1 antibodies only; (3) n=1 for both anti-GM3 antibodies and anti-GM4 antibodies; (4) n=1 for anti-GM3 antibodies only; (5) n=1 for anti-GQ1b antibodies only; (6) n=1 for anti-GM1 antibodies only; and (7) n=1 for both anti-GQ1b antibodies and anti-GT1a antibodies. For clinical classification, classical GBS was the most common in 31 (52%) cases, followed by facial palsy with distal paresthesia in 15 (25%) cases and paraparesis variant in 6 (10%) cases, MFS in 3 (5%) cases, paresthesia in 3 (5%) cases, and GBS-Miller Fisher syndrome overlap in 2 (3%) cases. Neurophysiological examination was performed in 52 cases, and the most common electrophysiological type was AIDP in 37 (71%) cases, followed by AMSAN in 5 (10%) cases, and AMAN in 3 (6%) cases, with 7 (13%) cases without neuropathy. For GBS treatment, 52 cases were treated with intravenous IgG, of whom 11 (21%) received concurrent ventilator-assisted ventilation, 4 (8%) received concurrent hormone therapy, 4 (8%) received concurrent plasma exchange therapy, 2 (4%) received hormonal therapy only, 2 (4%) presented with pure sensory impairment and were treated with gabapentin therapy only, and 4 (8%) received no therapy at all. Prognosis was reported in 54 cases, of whom 52 (96%) showed varying degrees of symptom improvement and only 2 (4%) had a poor prognosis (1 prolonged bed rest and 1 death).

DNA vaccines were the major vaccine type, provided in 37 (62%) cases, consisting of 33 (55%) vaccinated with ChAdOx1-S, 4 (7%) vaccinated with Ad26.COV2S, followed by RNA vaccine BNT162b2 in 22 (37%) cases. The 59 cases were divided into a DNA vaccine group and an RNA vaccine group by vaccine type. The two groups were compared in terms of mean age, sex, mean time from vaccination to the onset of symptoms, clinical subtype, and neurophysiological subtype (**Table 2**). The results showed that there were significant inter-group differences in the frequency of bilateral facial nerve palsy (P=0), clinical subtype (P=0.001), and CSF albuminocytologic dissociation (P=0.004). The frequency of bilateral facial nerve palsy was far greater in the DNA vaccine group than in the RNA vaccine group (76% vs. 18%). For clinical classification, facial palsy with distal paresthesia and CSF albuminocytologic dissociation had a higher frequency in the DNA group than in the RNA group (38% vs. 5% and 100% vs. 70%, respectively).

**Table 2:** Comparison of GBS between the DNA and RNA vaccine groups.

	GBS after DNA vaccine	GBS after RNA vaccine	P
**Age(years)**	55.32±12.18	52.14±23.58	0.561
Sex			0.261
Female	13(35%)	11(50%)	
Male	24(65%)	11(50%	
**Vaccination to symptom onset(days)**	12.24±5.19	12.68±8.21	0.823
Dose			0.327
1st 2nd	37(100%) 0	17(94%) 1(6%)	
Facial diplegia			0
No	9(24%)	18(82%)	
Yes	28(76%)	4(18%)	
Albuminocytological dissociation			0.004
No	0	6(30%)	
Yes	32(100%)	14(70%)	
Antiganglioside antibody			0.171
Negative	21(88%)	7(64%)	
Positive	3(12%)	4(36%)	
EMG			0.123
AIDP	25(78%)	12(63%)	
AMSAN	3(9%)	1(5%)	
AMAN	0	3(16%)	
Normal	4(13%)	3(16%)	
Clinical classification			0.001
Classic GBS	19(51%)	12(55%)	
Bilateral facial palsy with paresthesia	14(38%)	1(5%)	
Paraparetic	0	5(23%)	
Sensory GBS	2(5%)	1(5%)	
vMFS	0	3(14%)	
GBS / MFS overlap variants	2(5%)	0	

GBS, Guillain-Barré Syndrome; MFS, Miller-Fisher Syndrome; EMG, electromyography; AIDP, acute inflammatory demyelinating polyneuropathy; AMAN, acute motor axonal neuropathy; AMSAN, acute motor and sensory axonal neuropathy.

## Discussion

GBS is an immune-mediated demyelinating disease that often occurs after infection; *Campylobacter jejuni* and *Haemophilus influenzae* are the most common pathogens, although cytomegalovirus is the pathogen in some cases, while a few cases result from *Mycoplasma pneumoniae* (42). Vaccination is also a common cause of GBS, and post-vaccination GBS may occur after administion of any type of vaccine, including rabies vaccine, diphtheria-pertussis-tetanus vaccine, rubella vaccine, tetanus toxoid vaccine, hepatitis B vaccine, hepatitis A vaccine, and influenza vaccine, with onset mainly within two weeks after vaccination. An association between vaccines and GBS has never been proven for most of debated vaccines. For most vaccines, the debate between supporters and opponents of vaccination remains robust. Less than 1 case of GBS per million immunized persons might occur for these vaccines (43–45).The association between a vaccine and the increased incidence of GBS has been confirmed only for influenza vaccine so far, with 1–2 cases of GBS per 1 million doses of influenza vaccine (43–45). Molecular mimicry is often the primary pathogenic mechanism for vaccine-associated GBS. Specifically, the vaccine contains the same structure as gangliosides, and thus vaccinated individuals produce anti-ganglioside antibodies that attack neural autoantigens, thereby causing neurological damage and associated clinical symptoms.

With the widespread use of COVID-19 vaccines worldwide, many cases of post-vaccination GBS have been reported (7–41). Hill proposed that the criteria for assessing the causal relationship between clinical outcome and possible pathological injury consist of the following nine characteristics: strength of association, consistency, specificity, temporality, biological gradient, plausibility, coherence, experimental evidence, and analogy (46). The only evidence for the association of these cases with COVID-19 vaccines was temporality. The mean time from vaccination to symptom onset was 12.3 days in our review, which was consistent with the expected period of maximal immune response to the vaccine, while there was no evidence that the patient was subject to other infectious or autoimmune factors. Geographically, the cases reported so far are mainly in the UK and India, where DNA vaccines are the most common COVID-19 vaccines, and clinical trials have shown that DNA vaccines have a higher potential to trigger GBS (47). In addition to association with vaccine types, the geographical distribution of cases may also be closely associated with the severity of the national epidemic and the population vaccination rates. Moreover, the occurrence of GBS in a population is also strongly associated with the underlying incidence of GBS in that population, while the underlying incidence varies among different countries, and the role of host immunogenetic background in the development of GBS in different populations is associated with human leukocyte antigen (HLA) polymorphism in the population (48).

Several large-scale surveillance projects on the emergence of GBS after COVID-19 vaccination are already underway, involving national and international public health agencies (CDC, FDA, EMA, WHO) and neurological societies (49, 50). According to preliminary reports, 132 cases of GBS occurred after the administration of 13.2 million doses of the Johnson & Johnson vaccine in the United States and 227 cases of GBS occurred after the administration of 51 million doses of the AstraZeneca vaccine in Europe (47). Currently, the European Medicines Agency (EMA) and the Food and Drug Administration (FDA) have listed GBS as a side effect of Janssen, AstraZeneca, and Johnson & Johnson vaccines (49, 50). The Janssen, Johnson & Johnson, and AstraZeneca vaccines are all DNA vaccines, while our findings reveal a higher risk of GBS following DNA vaccination and a low incidence of GBS following RNA vaccination. Correlation analysis of the data from UK’s national vaccination database of novel coronavirus with hospitalization data shows that compared with before vaccination, the risk of GBS over the 28-day post-vaccination period increases by a factor of 2.04 with the AstraZeneca vaccine but does not increase with the Pfizer vaccine, with clinical trials observing no GBS cases following RNA vaccination (47). These findings suggest that the occurrence of GBS after COVID-19 vaccination is likely related to the nature of the vaccine.

DNA vaccines use a single recombinant replication-deficient chimpanzee adenovirus vector (ChAdOx1) to enter the cell and encode the spike protein of the SARS-CoV-2 virus, which is then exported to the cell surface to stimulate antibody and T-cell production. In mRNA vaccines, the mRNA molecules are included in lipid nanoparticles that allow the fusion with cellular membranes of host cells and hence the mRNA is released in the cytoplasm, where it is translated to build the spike protein. Antibodies against the spike protein may cross-react with peripheral nerve components (gangliosides) to cause GBS. However, contrary to expectations, GBS cases that occur after COVID-19 vaccination have a low positive rate of anti-ganglioside antibodies (20%), which is significantly lower than that (80%–90%) in other reported GBS cases, a discrepancy suggesting that gangliosides may not be the true antigenic target for GBS that emerges after COVID-19 vaccination (51). Molecular mimicry antigens may be structurally related to adenoviral vectors, which explains the relative safety of RNA vaccines (47). In addition, abnormal splice variants, contaminated proteins, or other components of the vaccine may all be sources of immune response in GBS, but its true antigenic targets remain to be further investigated.

Most cases in this study occurred after the first dose of vaccine, and the mean time from vaccination to the onset of neurological symptoms was 12.3 days, which was consistent with the time from vaccination to the onset of GBS symptoms. GBS occurred time-linked to the vaccination, the vaccination stimulates the production of T-cells and antibodies, which could cross-react with the structures of the nerve roots. It is to be noted that molecular mimicry requires a humoral response that requires 10-14d to develop (52). The cases mainly occurred in male patients.

We speculate that there are gender differences in the mechanism of immune response following COVID-19 vaccination because there are gender differences in the immune response after COVID-19 infection, with females producing a more potent immune response involving T cells and males producing more cytokines (53). The incidence of GBS following COVID-19 vaccination may be related to the different immune response mechanism. In the future, we need to collect their blood samples and monitor their immune response after vaccination to further confirm the hypothesis. The initial symptoms were paresthesia and limb weakness, and the classical GBS was the dominant clinical subtype, with a good prognosis. These characteristics were not significantly different from those of common GBS. In this study, 89% of the cases showed CSF albuminocytologic dissociation, which was higher than the 64% frequency of CSF albuminocytologic dissociation in the entire GBS population. The difference in the frequency of CSF albuminocytologic dissociation between the DNA vaccine group and the RNA vaccine group is presumably closely associated with the difference in the timing of CSF test and thus variable to some extent (47).

Of note, the present study observed that bilateral facial nerve palsy was generally the initial symptom of post-COVID-19 vaccination GBS and that the frequency of facial palsy with distal paresthesia was much higher than expected, with both bilateral facial nerve palsy and facial palsy with distal paresthesia being common in the DNA vaccine group. Pegat et al. analyzed all cases reported in the French pharmacovigilance database (June 29, 2021) and found that 23 (33%) of GBS cases following COVID-19 vaccination presented with bilateral facial nerve palsy, 21 (91%) of which occurred after vaccination with DNA vaccines—namely ChAdOx1-S (46% [20/44]) and Ad26.COV2S (33% [1/3])—accounting for 45% of all GBS cases following DNA vaccination (54). For GBS following DNA vaccination, facial involvement is more frequent and facial palsy with distal paresthesia is more common, suggesting that GBS may appear as a specific clinical subtype following DNA vaccination. This supports a possible causal relationship between COVID-19 vaccines and the syndrome, while it is still necessary to further elucidate this possible causal relationship and the underlying immunopathologic mechanism through prospective studies.

In contrast to common GBS cases that mainly involve axonal loss owing to infections by pathogens (*Campylobacter jejuni*, Mycoplasma), myelin involvement is more common in post-COVID-19 vaccination GBS (20, 24, 27, 55). Each clinical or neurophysiological subtype of GBS is closely associated with a type of serological antibody, and ideally there would be a specific antibody that explains a specific clinical subtype or neurophysiological subtype; however, thus far, only the association between anti-GQ1b/GT1a antibodies and MFS has been confirmed. Since the GBS cases following COVID-19 vaccination in this study generally tested negative for antibodies, we were unable to analyze the correlation between antibodies and clinical subtypes (48). However, the negative serology in the majority of the cases suggested that the dominant neurophysiological subtype of post-COVID-19 vaccination GBS is likely to be AIDP; this is because (1) anti-ganglioside antibodies are highly selective in attacking axons and are considered to be a biomarker of axonal injury rather than demyelination and (2) anti-ganglioside antibodies (GM1, GM1b, GD1a) are common in the serum of cases with the AMAN subtype of GBS, while the AIDP subtype was largely serologically negative for these antibodies, consistent with our expectation (55, 56). In short, the present results suggest that the neurophysiological subtypes of GBS following COVID-19 vaccination are indeed dominated by AIDP (55, 56).

The present study summarized these cases in the hope that they may reflect the clinical features of post-COVID-19 vaccination GBS and thus shed some light on the underlying pathophysiological mechanism. The study was subject to the limitations of passive reporting systems and presumptive case definition. Besides, spontaneous reports contain incomplete information, so it is not yet possible to establish a reliable causal relationship between COVID-19 vaccination and GBS occurrence by relying on these cases alone. As mentioned earlier, the risk of post-vaccination GBS has been well documented only for influenza vaccination, with an increase of 1–2 cases of GBS per 1 million doses of influenza vaccine. Consequently, the risk of post-influenza vaccination GBS was used as a reference in one study, which observed that the risk of post-COVID-19 vaccination GBS was much lower than the reference, namely approximately 0.26 cases of GBS per 1 million doses of COVID vaccine, which suggests that the COVID-19 vaccine is safe and does not increase the risk of GBS (57, 58). The GBS/CIDP Foundation also states that there is no clear evidence that COVID-19 vaccination increases the risk of GBS (59). There is early evidence that the administration of COVID-19 vaccination, specifically the Pfizer vaccine, among patients with history of GBS is not associated with a significant risk of relapse (60). Therefore, we continue to advocate for COVID-19 vaccination, but we recommend population surveillance to facilitate timely detection and management of GBS that may occur after COVID-19 vaccination. As a clinical practice, we currently intend to highlight a precaution in patients with GBS following COVID-19 vaccination for revaccination with another mechanism and revaccination should preferably be administered during a remission phase of the disease (34).

## Conclusion

This review suggests that GBS following COVID-19 vaccination may be associated with the first dose of the vaccine, especially DNA vaccines. It is similar to regular GBS in terms of main clinical symptoms and CFS characteristics but with a higher rate of facial involvement and a lower positive rate of anti-ganglioside antibodies. It is critically important for physicians to rapidly recognize and early diagnosis of this type of GBS. The data is insufficient to determine the link between GBS and COVID-19 vaccination. Larger observational studies are required to adequately determine the link and the pathogenesis of GBS, particularly as new strains of the virus emerge and new vaccines are developed. The overwhelming evidence suggests that the benefits of vaccination far outweigh the slightly increased risk of GBS. The prognosis for GBS following COVID-19 vaccination is generally good, so regardless of whether there is a causal link between GBS and COVID-19 vaccination, GBS cannot be considered a reason to avoid the administration of recommended vaccines.

## Author contributions

Conceptualization: all authors; methodology, formal analysis, and investigation: MY; SN; writing—original draft preparation: MY, YM; writing—review and editing: YM, YQ. All authors contributed to the article and approved the submitted version
